# MiR-34a-5p promotes multi-chemoresistance of osteosarcoma through down-regulation of the DLL1 gene

**DOI:** 10.1038/srep44218

**Published:** 2017-03-10

**Authors:** Youguang Pu, Fangfang Zhao, Haiyan Wang, Shanbao Cai

**Affiliations:** 1 Cancer Epigenetics Program, Anhui Cancer Hospital, West Branch of Anhui Provincial Hospital, Anhui Medical University, Hefei 230031, Anhui, China; 2 Department of Clinical Geriatrics, Anhui Provincial Hospital, Anhui Medical University, Hefei 230031, Anhui, China; 3 Department of Orthopedic Surgery, Anhui Cancer Hospital, West Branch of Anhui Provincial Hospital, Anhui Medical University, Hefei 230031, Anhui, China

## Abstract

MiR-34a-5p has been implicated in the tumorigenesis and progression of several types of cancer. However, the role of miR-34a-5p in osteosarcoma (OS) remains largely unknown. This study was performed in two multi-chemosensitive (G-292 and MG63.2) and two resistant (SJSA-1 and MNNG/HOS) OS cell lines. MiR-34a-5p promotes OS multi-chemoresistance via its repression of the Delta-like ligand 1 (DLL1) gene, the ligand of the Notch pathway, and thus negatively correlates with OS chemoresistance. The siRNA-mediated repression of the DLL1 gene suppressed cell apoptosis and de-sensitized G-292 and MG63.2 cells, while overexpression of DLL1 sensitized SJSA-1 and MNNG/HOS cells to drug-induced cell death. In agreement with the changes in the drug-induced cell death, the activity of the ATF2/ATF3/ATF4 signaling pathway was significantly altered by a forced reversal of miR-34a-5p or DLL1 levels in OS cells. DLL1 is a target of miR-34a-5p and negatively regulates the multi-chemoresistance of OS. This study suggested that miR-34a-5p, DLL1 and the ATF2/ATF3/ATF4 signaling pathway-associated genes are the potential diagnostic and/or therapeutic targets for an effective chemotherapy of OS. Our results also provide novel insights into the effective chemotherapy for OS patients.

MicroRNAs (miRNAs) are a large group of small non-coding regulatory RNA molecules that have been shown to play vital roles in various biological processes^1^. Their dysregulation has been associated with the development of cancer. The abnormal expression of miRNAs in cancer contributes to every event of tumor biology^2^,^3^, including chemoresistance^4^, which remains a major hinder to effective treatment^5^. The multi-chemoresistance varies dramatically among the cancer patients, even in the different cancer lesions or different regions of the same lesions within a single patient^6^. Despite of intensive studies, our knowledge of the cancer multi-chemoresistance remains very poor due to the various inducers^7^,^8^. To date, much effort has been exerted in investigating the role of miRNAs in the chemoresistance of different cancers. The eminent examples for bladder cancer chemoresistance are miR-30d, miR-181, miR-199a-5p^9^ and miR-193a-3p^10^,^11^. In hepatocellular carcinoma (HCC) cells, the DNA methylation-regulated miR-193a-3p dictates the 5-FU resistance *via* repressing SRSF2 expression in particular^10^. Moreover, in bladder cancer, miR-193a-3p positively regulates the multi-chemoresistance *via* repression of three target genes, SRSF2, HIC2 and PLAU^11^. In addition, over-expression of miR-21 in colorectal cancer tissues is less sensitive to 5-FU^12^. Inhibition of miR-130a could overcome cisplatin (CDDP) resistance by regulating the MDR1/P-gppathway^13^. Of note, miR-140 is involved in the osteosarcoma (OS) chemoresistance by decreased cell proliferation via G1-and G2-phase arrest^14^.

As the well-studied miRNAs, the members in the miR-34 family (miR-34a, mi-R34b and miR-34c) show high sequence similarities^15^ and are directly regulated by the transcription factor p53^16^,^17^,^18^. For example, miR-34a negatively regulates Delta-like ligand 1 (DLL1) of the Notch pathway and thus down-regulates cell proliferation by inducing apoptosis and neural differentiation in medulloblastoma cells. In gliomas, miR-34a targets multiple oncogenes, such as c-Met, CDK6, Notch1, and Notch2, which suggests that miR-34a might act as a therapeutic agent for brain tumors^19^. Furthermore, miR-34a-5p, derived from miR-34a, has been found to inhibit cell invasion and migration^20^,^21^,^22^,^23^, which suggests that miR-34a-5p might be involved in inhibiting tumor recurrence.

OS is the most common malignant primary bone tumor in children and adolescents^9^,^24^, and the mechanism for OSchemoresistanceremains largely unknown. In the present study, we performed a RNA-seq-based-omic analysis to detect the differentially expressed genes in two multi-chemosensitive (G-292 and MG63.2) versus two resistant (SJSA-1 and MNNG/HOS) OS cell lines. We showed that miR-34a-5p promoted OS multi-chemoresistance *via* repression of the DLL1 gene, a new target of miR-34a-5p. We further performed a systematic analysis of the DLL1 gene for its role in and mechanisms by which the multi-chemoresistance of OS cells is regulated.

## Results

### DLL1 is a negative regulator of OS multi-chemoresistance

Our previous report suggested that G-292/MG63.2 and SJSA-1/MNNG/HOS are multi-chemosensitive and multi-chemoresistant OS cell lines, respectively^25^. To identify the mechanisms that govern the multi-chemoresistance of OS cells, we performed an RNA-seq-based miR-omic analysis of G-292, MG63.2 and SJSA-1 cells (GEO accession number: GSE89930). The results showed that a dozen of miRNAs were differentially expressed in the multi-chemoresistant OS cells SJSA-1 and the multi-chemosensitive OS cells G-292 and MG63.2 (Additional File 1A). Among these miRNAs, miR-34a-5p was reported to be involved in the multi-chemoresistance of colorectal cancer^26^. We thus selected miR-34a-5p as our target for investigating its roles in OS chemoresistance.

**Figure 1:**
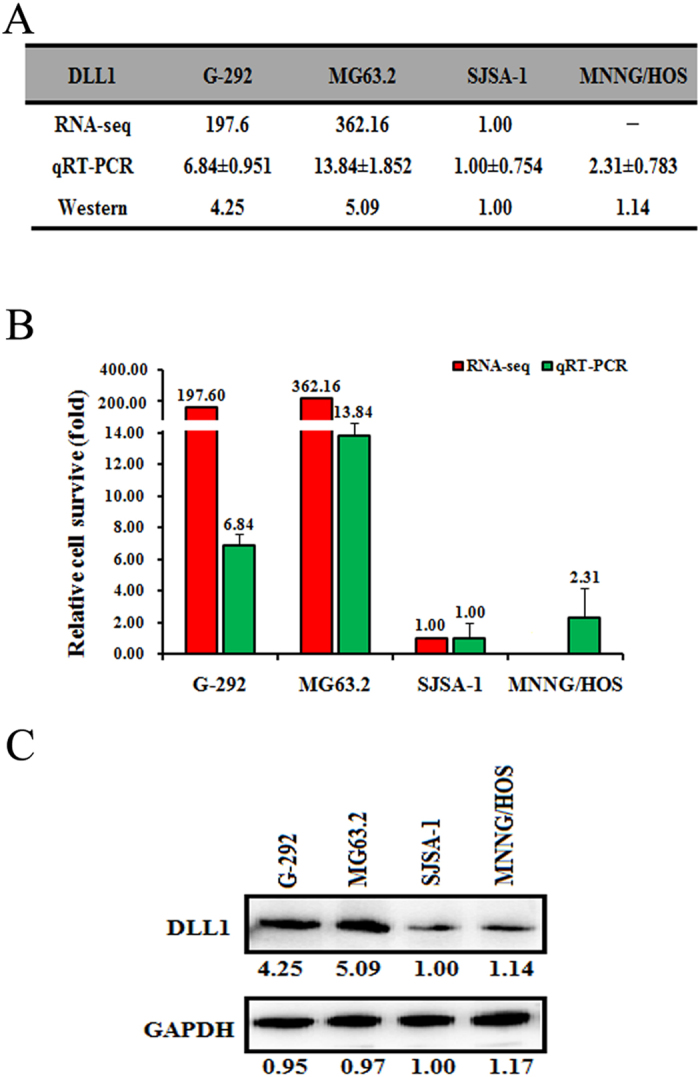
DLL1 is a negative regulator of OS multi-chemoresistance. The relative level (fold) of the DLL1 gene is also summarized in table (**A**), by miR-seq and qRT-PCR analyses in plot (**B**), analyzed by western analysis (**C**). “-” Indicates no detection in the omic analysis.

A given miRNA usually suppresses the expression of various target genes and thus regulates related pathways. The RNA-seq based miR-omic analysis also revealed several differentially expressed genes between different OS cell lines, which might suggest their relationship with OS multi-chemoresistance. To find the target genes of miR-34a-5p, we first predicted the target genes of miR-34a-5p based on the following websites: TargetScan (http://www.targetscan.org/), miRDB (http://mirdb.org/miRDB/) and microRNA.org (http://www.microrna.org/microrna/getMirnaForm.do). Several common genes were found(Additional File 1B), and some of them were then subject to testing the expression of both mRNA and protein levels between G-292/MG63.2 and SJSA-1/MNNG/HOS cells. At last, we found several potential target genes of miR-34a-5p, such as CD117^25^, AGTR1 and DLL1. Here we select DLL1 as our target for further studies. The expression of DLL1 was higher in G-292 and MG63.2 than SJSA-1 and MNNG/HOS cells at both mRNA (RNA-seq-based miR-omic: 197.60:362.16:1.00 in G-292, MG63.2 and SJSA-1, respectively, and the qRT-PCR analysis: 6.84:13.84:1.00:2.31 in G-292, MG63.2, SJSA-1 and MNNG/HOS, respectively; Fig. 1A and B) and protein level (4.25:5.09:1.00:1.14 in G-292, MG63.2, SJSA-1 and MNNG/HOS, respectively; Fig. 1C).

### The DLL1 gene is a target of miR-34a-5p in OS cells

**Figure 2:**
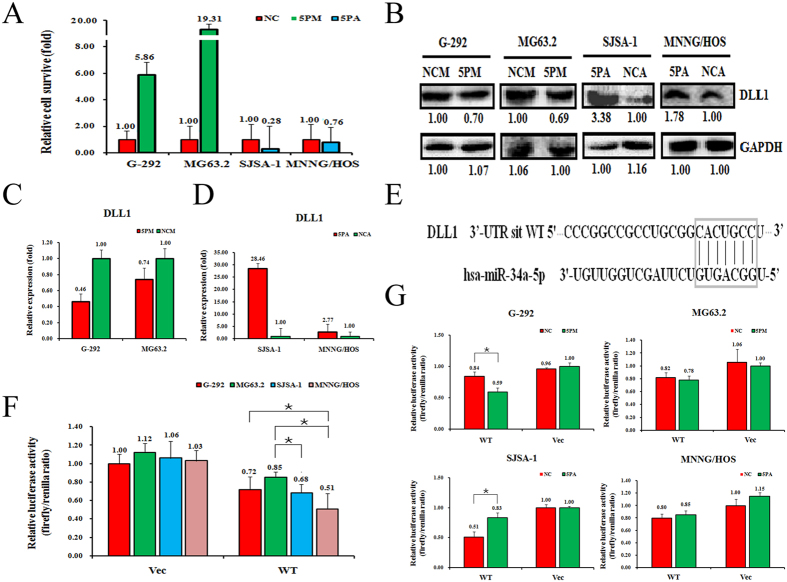
DLL1 is a target of miR-34a-5p in OS cells. The level of miR-34a-5p (**A**) and DLL1 protein (**B**) and mRNA (**C**) and (**D**) in the miR-34a-5p mimic (5PM)-transfected G-292 and MG63.2 cells and the miR-34a-5p antagomiR (5PA)-transfected SJSA-1 and MNNG/HOS cells versus the corresponding negative control (NCM or NCA) determined by western analyses or qRT-PCR. (**E**), the sequences in the UTR region of the DLL1 gene targeted by miR-34a-5p (in the box). (**F**) and (**G**), the relative luciferase activity (fold) of the reporter with wild-type (WT) DLL1-UTR or with no UTR (Vec) was determined in the miR-34a-5p mimic (in G-292 and MG63.2), antagomiR (in SJSA-1 and MNNG/HOS) or corresponding mock-transfected OS cells. The Renilla luciferase activity of a co-transfected control plasmid was used to control the transfection efficiency. The representative results from three independent experiments are shown. Error bars represent the s.e.m. *P-value < 0.05; by Student’s *t*-test.

The miR-34a-5p level was higher in SJSA-1 and MNNG/HOS cells than G-292 and MG63.2 cells. DLL1 expression negatively correlated with miR-34a-5p levels. To evaluate whether DLL1 is an authentic target of miR-34a-5p, we determined DLL1 levels in the miR-34a-5p mimic-transfected G-292 and MG63.2 cells and the antagomiR-transfected SJSA-1 and MNNG/HOS cells versus the corresponding NC (scramble sequence control)-transfected cells. The transfection of miR-34a-5p mimic in G-292 and MG63.2 increased its expression by 5.86% and 19.21%, respectively, whereas the transfection of miR-34a-5pantagomiR in SJSA-1 and MNNG/HOS significantly decreased its level to 28% and 76%, respectively (Fig. 2A). In accordance with the changes of the miR-34a-5p level, miR-34a-5p mimic transfection decreased the DLL1 protein level to 70% and 69% (Fig. 2B) and the mRNA level to nearly 46% and 74% (Fig. 2C) of that in the corresponding NC-transfected G-292 and MG63.2 cells, respectively. As expected, miR-34a-5p antagomiR transfection increased the protein level of DLL1 to 3.38-folds and 1.78-folds (Fig. 2B) and the mRNA level by 28.46- and 2.77-folds in SJSA-1 and MNNG/HOS cells, respectively (Fig. 2D).

To further confirm that DLL1 is a target of miR-34a-5p, we cloned the wild-type DLL1 gene downstream of the Renilla luciferase gene of the pGL3-control vector (Promega) to create pGL3-DLL1 UTR WT (Fig. 2E). The constructs pGL3-DLL1 UTR WT and pGL3 enhancer control were transfected into G-292, MG63.2, SJSA-1 and MNNG/HOS cells to determine the function of miR-34a-5p in different OS cells. The pGL3-DLL1-UTR WT had comparable luciferase activity in the four cells (Fig. 2F). Furthermore, the luciferase activity of pGL3-DLL1-UTR WT but not the control constructs was reduced in the miR-34a-5p-mimic-transfected G-292 and MG63.2 cells and increased in the miR-34a-5p-antagomiR-transfected SJSA-1 and MNNG/HOS cells (Fig. 2G). Taken together, DLL1 is indeed a target of miR-34a-5p and may conduct miR-34a-5p’s effect on OS chemoresistance.

### DLL1 expression negatively correlates with the effect of miR-34a-5p on OS chemoresistance

**Figure 3:**
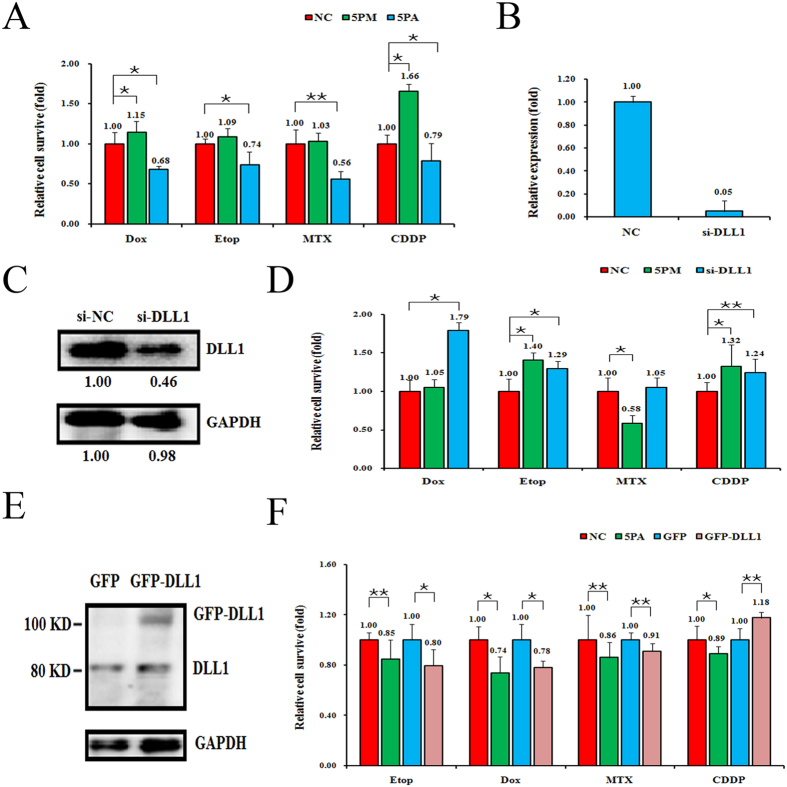
The effects of a forced reversal of the miR-34a-5p or DLL1 levels on the chemoresistance of G-292 and SJSA-1 cells. The IC_50_-dosed drug-induced cell death of G-292 and SJSA-1 cells transfected with the miR-34a-5p mimic (5PM) or antagomiR (5PA) versus the corresponding negative control (NC) assayed 72 hr post-treatment (**A**). The levels of DLL1 mRNA by qPCR analysis in the siRNA- transfected G-292 cells versus the si-NC (**B**). The DLL1 protein level (western blot analysis) in the siRNA-transfected versus the NC-transfected G-292 cells (**C**). The relative cell survival of the G-292 cells transfected by siRNA over the NC-transfected G-292 cells 72 hr after treatment of the IC_50_ dosed drugs (**D**). The DLL1 protein level (western blot analysis) in the GFP-tagged overexpression construct-transfected versus the NC-transfected SJSA-1 cells (**E**). The relative cell survival of the SJSA-1 cells transfected with miR-34a-5p antagomiR (5PA) over the NC-transfected SJSA-1 cells assayed 72 hr post-treatment of the IC_50_ dosed drugs (**F**). (NC was normalized, *P-value < 0.05; **P-value < 0.01).

To explore the role of DLL1 in OS chemoresistance, we transfected G-292 cells with the miR-34a-5p mimic and the SJSA-1 cells with miR-34a-5p antagomiR and compared the cell death triggered by an IC_50_-dosed drug. The transfection of miR-34a-5p mimic in G-292 cells significantly increased drug resistance to some extent, with CDDP mostly to 1.66-fold, whereas the transfection of miR-34a-5p antagomiR in SJSA-1 cells decreased the chemoresistance for all four drugs (Fig. 3A). We further transfected si-DLL1 into G-292 cells to evaluate its effect on multi-chemoresistance. The transfection of si-DLL1 indeed decreased the level of DLL1 at both the mRNA (0.05:1.00) and protein level (0.46:1.00) compared with the corresponding control cells (Fig. 3B and C). Following the decrease in the DLL1 level in G-292 cells upon transfection of miR-34a-5p mimic, the cell survival rate was increased byapproximately 5%-40% with the addition of the four drugs, excluding MTX (Fig. 3D). Furthermore, the transfection of si-DLL1 increased the cell survival rate with the addition of all four drugs (Fig. 3D). The results correlated well with the negative regulation of the multi-chemoresistance of OS cells. Conversely, the transfection of a GFP-tagged DLL1 expression construct raised the DLL1 protein level in SJSA-1 cells (Fig. 3E). The resultant chemoresistance was significantly decreased for all four drugs, excluding CDDP. As expected, the control cells with the transfection of only GFP protein showed a marginal effect on the chemoresistance of SJSA-1 (Fig. 3F). Similar cases in which the chemoresistance was significantly decreased for all four drugs were also found with the transfection of miR-34a-5p antagomiR in SJSA-1 cells (Fig. 3F).

**Figure 4:**
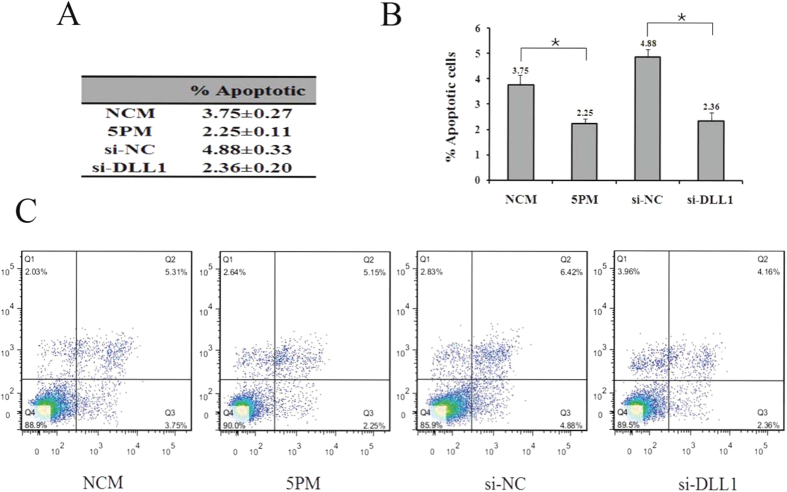
The effects of the forced reversal of both miR-34a-5p and DLL1 levels on the apoptosis of G-292 cells by FACS analysis in the plot and in the original (**A**), (**B**)and (**C**). ((NC was normalized, *P-value < 0.05).

In agreement with its negative effect on chemoresistance (Fig. 3D and F), a siRNA-mediated DLL1 repression decreased the apoptotic rate from 4.88% to 2.36%, and a similar effect was found in the miR-34a-5p-mimic-transfected G-292 cells (Fig. 4A,B and C). Taken together, both DLL1 and miR-34a-5p contribute substantially to the OS chemoresistance to Dox, Etop, MTX and CDDP.

### The ATF2/ATF3/ATF4 signaling pathway might be involved in OS multi-chemoresistance

**Figure 5:**
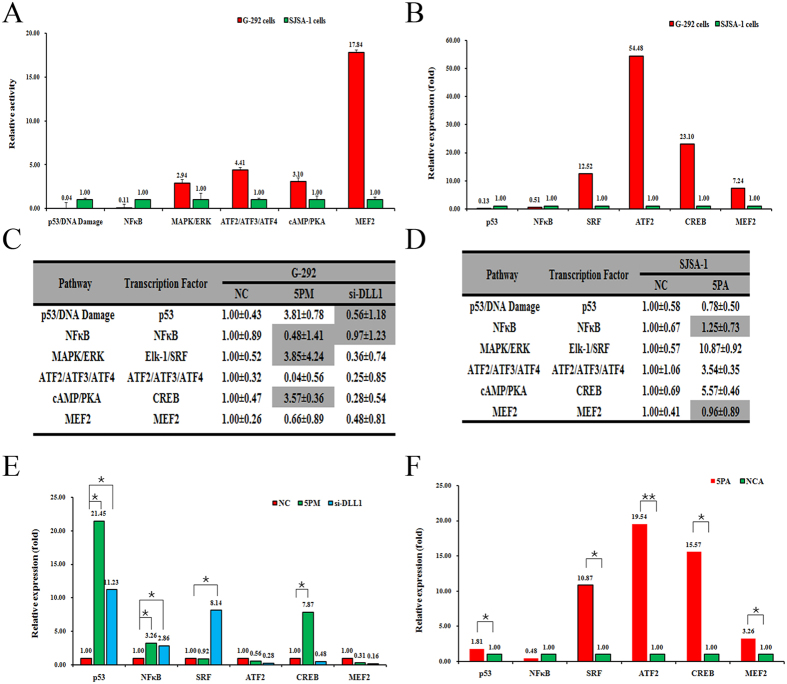
The effects of the forced reversal of both miR-34a-5p and DLL1 levels on the activity of the signaling pathways in SJSA-1 versus G-292 cells. The relative activities of the six indicated pathways in SJSA-1 versus G-292 cells (**A**) and the relative expression ratio of the six transcription Factors (**B**). The relative pathway activities in the DLL1 siRNA- or miR-34a-5p mimic- (5PM) versus the NC-transfected G-292 cells (**C**). The relative pathway activities in the miR-34a-5p antagomiR (5PA) versus the NC transfected SJSA-1 cells (**D**).The relative expression ratio of the six transcription factors in the DLL1 siRNA- or miR-34a-5p mimic- (5PM) versus the NC-transfected G-292 cells (**E**). The relative expression ratio of the six transcription factors in the miR-34a-5p antagomiR- (5PA) versus the NC-transfected SJSA-1 cells (**F**). (NC was normalized, *P-value < 0.05; **P-value < 0.01).

To gain further mechanistic insights into the roles of miR-34a-5p/DLL1 axis, we used the Cignal reporter finder assay to compare the activities of 17 signaling pathways in G-292 versus SJSA-1cells^27^. The following six cancer-related signaling pathways: p53/DNA damage, NF-кB, MAPK/ERK, ATF2/ATF3/ATF4, cAMP/PKA and MEF2 were selected, whose activities differed by more than two-fold. Among them, two pathways, p53/DNA damage and NF-кB, showed higher activities in SJSA-1 cells, whereas the other four pathways, MAPK/ERK, ATF2/ATF3/ATF4, cAMP/PKA and MEF2, showed higher activities in G-292 cells (Fig. 5A). These differences might imply a role in OS chemoresistance. Among them, two pathways, p53/DNA damage and NF-кB, showed higher activities in SJSA-1 cells, whereas the other four pathways, MAPK/ERK, ATF2/ATF3/ATF4, cAMP/PKA and MEF2, showed higher activities in G-292 cells (Fig. 5A). The expression of the corresponding transcription factors correlated well with the differential activity of these signaling pathways (Fig. 5B). We then compared the activities of all these pathways in miR-34a-5p mimic-transfected G-292 cells or miR-34a-5p antagomiR-transfected SJSA-1 cells. The activity of the p53/DNA damage pathway was elevated in the miR-34a-5p mimic-transfected G-292 cells, whereas it was reduced in the miR-34a-5p antagomiR-transfected SJSA-1 cells (Fig. 5C–F). By contrast, the activity of the ATF2/ATF3/ATF4 pathway was reduced in the miR-34a-5p mimic-transfected G-292 cells but increased in the miR-34a-5p antagomiR-transfected SJSA-1 cells (Fig. 5C–F). The activities of the above two pathways negatively correlated with miR-34a-5p’s promoting effect on OS chemoresistance, indicating their association with OS chemoresistance. We further compared the pathway activities in DLL1 siRNA versus the mock siRNA-transfected G-292 cells. Although a slight decrease on the activities of the DNA damage and NF-кB pathways was observed, the other three pathways, including ATF2/ATF3/ATF4, were significantly repressed by DLL1 siRNA, indicating the correlation of these three pathways with OS chemoresistance. Taken together, only the ATF2/ATF3/ATF4 pathway was validated to be involved in the OS chemoresistance mediated by miR-34a-5p.

### The DLL1 expression negatively correlates with the level of miR-34a-5p in the tumor xenografts of nude mice

**Figure 6:**
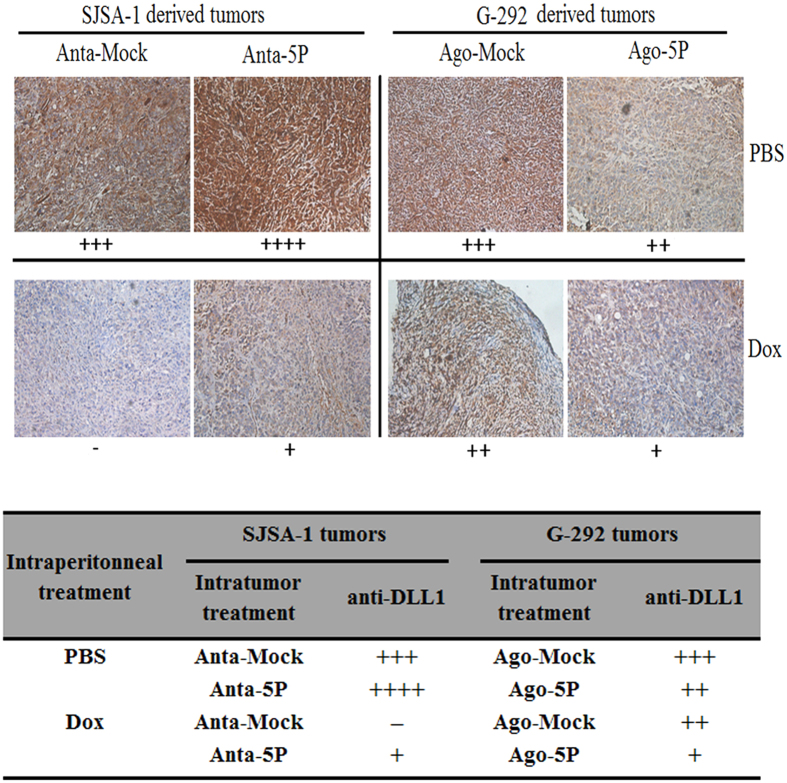
The DLL1 level in themiR-34a-5p agomiR-injected G-292 and the miR-34a-5p antagomiR-injected SJSA-1 tumor xenograft versus the NC-injected tumor xenografts determined by immunohistochemical staining. The G-292 and SJSA-1 tumor tissues from each group were fixed on one slide and immunostained for each indicated antibody. The levels of DLL1 protein in each group were determined by immunostaining and are summarized in the table (Magnification: 200×).

Recently, miR-34a-5p was shown to promote Dox chemoresistance of OS in tumor xenografts of nude mice via repressing its target gene CD117^25^. In the present study, we semi-quantified the levels of DLL1 protein in the same set of the tumor tissues in mice *via* immuno-histological analysis. The intratumoral injection of miR-34a-5p’s agomiR into G-292 decreased DLL1 expression. By contrast, the injection of miR-34a-5p’s antagomiR into SJSA-1 increased DLL1 expression in Dox- or PBS-treated mice (Fig. 6). The results further confirmed that miR-34a-5p has a positive effect on both growth and chemoresistance of OS cell-derived tumor xenografts in nude mice.

## Discussion

As a well-studied miRNA, miR-34a has been studied in several types of cancer, including Ewing’s sarcoma^28^ and colorectal cancer^27^. Several direct targets have been found for miR-34a, including Notch, c-Myc, c-Met, and c-Kit^29^. MiR-34a targets Notch1 and Notch2 in glioblastoma and medulloblastoma^19^. MiR-34a suppresses invasion via repression of Notch1 and Jagged1 in cervical carcinoma and choriocarcinomacells^30^. Moreover, much effort has been exerted to reveal the roles of miR-34a in cancer chemoresistance^31^,^32^,^33^. In this study, we showed that miR-34a-5p is also involved in the multi-chemoresistance of OS. MiR-34a-5p is up-regulated in multi-chemoresistant (SJSA-1 and MNNG/HOS) compared with multi-chemosensitive (G-292 and MG63.2) OS cell lines (Figs 1 and 2). Bisulfite Sequencing PCR (BSP) assay showed that the average methylation ratio of the miR-34a-5p promoter is comparable between SJSA-1 and G-292 cells (Additional File 2), which indicates that there are other factors that differentiate the expression of miR-34a-5p in SJSA-1 and G-292 cells. We next performed an RNA-seq analysis of G-292, MG63.2 and SJSA-1 cell lines and found a group of genes that were differentially expressed, including the DLL1 gene, which negatively correlates with chemoresistance (Additional File 1B). Both the role and mechanism of the DLL1 gene in the context of OS chemoresistance were systematically investigated in cultured cells and tumor xenografts in nude mice.

The DLL1 gene encodes one of the ligands in the Notch signaling pathway, which is highly conserved to regulate cell fate determination, stem cell self-renewal, proliferation and apoptosis^34^. The Notch pathway is composed of four receptors (Notch1–4) and five ligands (Jag1–2, DLL1, DLL3-4). Upon ligand binding, the activation of Notch is governed by a two-step proteolytic cleavage by ADAMS family protease and then γ-secretase to release the intracellular domain of Notch (ICN), which is subject to forming a complex with *Mastermind- like* 1 (MAM1)^35^. Recent studies have demonstrated the oncogenic potential of Notch as well as its constitutive activation in different type of cancers, such as human T-cell acute lymphoblastic leukemia^36^, non-small cell lung cancer^37^, ovarian carcinomas^38^, and pancreatic cancer^39^. Meanwhile, Notch1 signaling was suggested to be activated in human OS and may be associated with tumor invasion and metastasis^40^,^41^,^42^. The metastatic OS cell lines have higher levels of Notch 1, Notch 2, DLL1 and the Notch-induced gene Hes1^43^. Notch2, Jagged1, HEY1, and HEY2 were over-expressed in OS biopsy specimens whereas Notch1 and DLL1 were down-regulated^44^. In addition, the miR-34 cluster was inversely correlated with invasiveness in a small panel of OS tumors, suggesting that the miR-34 family members may be responsible for regulating Notch expression^42^. Here, we showed that DLL1 expression is elevated in multi-chemoresistant OS cell lines. DLL1’s involvement in OS appears more complicated, as it can be up- or down-regulated in different OS cell lines.

**Figure 7:**
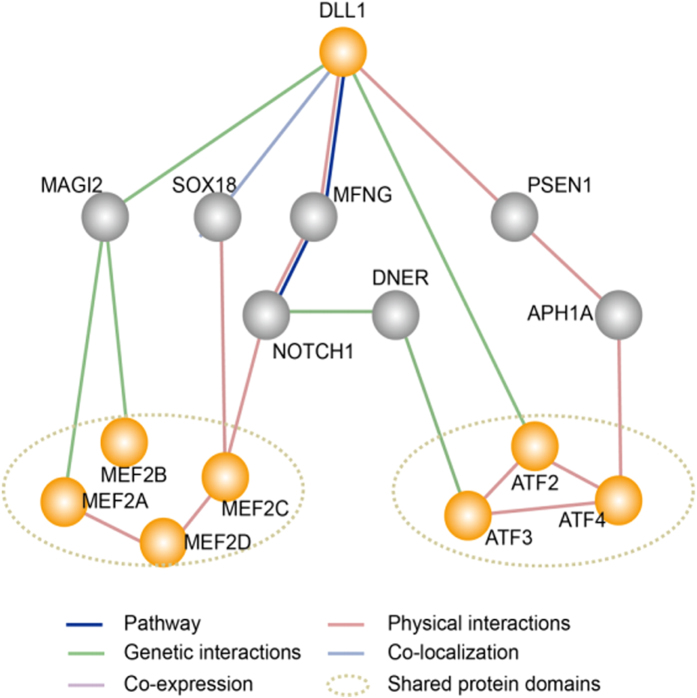
A simplified interaction map was analyzed between the target gene (DLL1) with the TGFβ, Myc/Max and ATF2/ATF3/ATF4 pathways by GeneMANIA (http://genemania.org/). Orange nodes represent the purposed genes. Gray nodes represent the genes that related with purposed genes through GeneMANIA method. The web-based interface searches gave a large set of functional association data to return related genes based on available genomic and proteomic data. The association data include protein, DNA and genetic interactions, pathways, gene and protein expression data, phenotypic screens and shared protein domains.

For further mechanistic understanding, we compared the activity of cancer-related signaling pathways, including Notch, in G-292 and SJSA-1 cells. No significant difference in the Notch pathway was found, even though DLL1 is a Notch ligand. Alternatively, we found that the ATF2/ATF3/ATF4 signaling pathway is involved in the DLL1-mediated repression of OS chemoresistance. We therefore further searched for the interactions between DLL1 and the master transcription factor gene for the ATF2/ATF3/ATF4 signaling pathway (Fig. 7) using GeneMANIA^45^. In total, 16 interacting genes of DLL1 were found in both GeneMANIA and the UniHIdatabase^5^. Then, these 16 genes were subjected to search for the relationships with the ATF2/ATF3/ATF4 transcription factors. Based on literature mining, a sophisticated connection network concerning a series of interactions was generated (Fig. 7). The DLL1 gene showed a direct genetic interaction with ATF2. In addition, DLL1 regulates ATF4 via a physical interaction with PSEN1, whereas DLL1 affects ATF3 via the interaction network of DLL1-MFNG-NOTCH1-ATF3. Notably, the PSEN1 gene regulates miR-193a-3p’s promoting effect on multi-chemoresistance in bladder cancer^46^. Notch1 plays a central role in the interplay of DLL1 with ATF2/ATF3/ATF4 and MEF signaling pathways. Thus, Notch1 may be the key player for the regulation of DLL1 in the ATF2/ATF3/ATF4 pathway. Further studies are needed for elucidating the fine mechanisms of the DLL1-regulated OS multi-chemoresistance. In addition, the links between the observations found in this study and the clinical practice for better OS chemotherapy warrant further exploration. Notably, a previous report suggested that the expression of miR-34a reduces drug resistance by targeting CD117 in colorectal cancer^26^, which is contradictory to the promoting effect of miR-34a-5p in OS drug resistance. This conflict might reflect the complicated regulatory mechanism of the miR-34a-mediated cancer drug resistance in different cells.

## Conclusion

In summary, we demonstrated that DLL1 is a target of miR-34a-5p and negatively regulates the OS multi-chemoresistance. The ATF2/ATF3/ATF4 signaling pathway might be involved in the miR-34a-5p’s promoting effect on OS chemoresistance. Targeting miR-34a-5p as well as its target gene DLL1 through novel therapeutics may provide a valuable strategy to overcome OS chemoresistance.

## Methods

### Cell lines and transfection

Cell lines—G-292 (ATCC NO. CRL-1423), SJSA-1 (ATCC NO. CRL-2098) and MNNG/HOS (ATCC NO. 1547) were purchased from the ATCC. Another cell line, MG63.2, derived from MG63 was kindly donated by Dr. Luu from the University of Chicago^47^. The four cell lines cells were cultured in Dulbecco’s modified Eagle’s medium (DMEM) (Gibco, USA) supplemented with 10% fetal bovine serum (Gibco, USA) in a humid atmosphere containing 5% CO_2_ at 37 °C.

### RNA-seq analysis

RNA-seq analysis was performed by BGI-Tech (Shenzhen, China), the method has been described in our previous report^48^. The raw data were deposited to the GEO dataset with the accession code of GSE89930.

### Cell reagents

The *Homo sapien* miR-34a-5pmimics, miR-34a-5p antagomiR, DLL1 siRNA and their corresponding scramble sequences (negative control, NC) and the transfection kit were supplied by Guangzhou Ribobio, China. The mammalian expression constructs for DLL1 (EX-Y3540-M98-5) with GFP tag were supplied by Guangzhou Fulengen, China. Transfection of the ribonucleic acid reagents or plasmids mentioned in this paper and the reporter plasmids in Cignal Finder™ Pathway Reporter Arrays (SABiosciences, USA) has been described previously^25^. The sequences used in this study are as follows:

si-DLL1: 5′-CCAUAAGCCCUGCAAGAAU dTdT-3′ 3′-dTdT GGUAUUCGGGACGUUCUUA-5′ hsa-miR-34a-5p antagomiR: 5′ACAACCAGCUAAGACACUGCCA 3′ mimics: sense 5′UGGCAGUGUCUUAGCUGGUUGU 3′ antisense 5′ACAACCAGCUAAGACACUGCCA 3′

### Chemoresistance profiling (IC_50_ determination)

Clinical grades of the following drugs were used (NCI Dictionary of Cancer Terms, http://www.cancer.gov/dictionary), Dox (Haizheng, Zhejiang, China); Etop (Hengrui, Jiangsu, China); MTX (Lingnan, Guangdong, China) and CDDP (Haosen, Jiangsu, China)^11^,^49^,^50^.

### RNA analysis

Total RNA was extracted using TRIzol (Vazyme). For the mRNA analysis, the cDNA primed by oligo-dT was made with RT reagent kit (Tiangen, China), and the mRNA level of DLL1 was quantified by a duplex-qRT-PCR analysis where the TaqMan probes with a different fluorescence for β-actin (Shing Gene, China) were used in the FTC-3000P PCR instrument (Funglyn, Canada). The miRNA expression level was normalized using U6 small nuclear RNA (HmiRQP9001) as an internal control, as previously described^51^. Using the 2−^ΔΔ^Ct method, the β-actin level was normalized before comparing the relative level of the target genes. The sequences of primers and probes used for the qRT-PCR analysis are as follows:

HDLL1 F: 5′-GTTCAGCAACCCCATCCG-3′ HDLL1 R: 5′-TCTGGGTTTTCTGTTGCGAG-3′ HDLL1 probe: 5′-CY5- CCTGGCCGGGCACCTTCTCTC-3′ HTP53 F: 5′-GACGGAGGTTGTGAGGCG-3′ HTP53 R: 5′-CTTCCACTCGGATAAGATGCTG-3′ HTP53 probe: 5′-CY5-CCACCATGAGCGCTGCTCAGATAGC-3′ HNFKB F: 5′-CAATGCCCTTTTCGACTACG-3′ HNFKB R: 5′-GGTGGATGATTGCTAAGTGTAAGA-3′ HNFKB probe: 5′-CY5- ACGTGAAGATGCTGCTGGCCGTC-3′ HSRF F: 5′-TACCCACCGCTGAAACGC-3′ HSRF R: 5′-CGGAAGGACTAGGTGTCTGATC-3′ HSRF probe: 5′-CY5-CGAAGTAGTGCTGACCCTGCACTCAATC-3′ HATF2 F: 5′-ATGAGTTGGCGAGTCCATTTG-3′ HATF2 R: 5′-TGTTTCTACAACAGAAGGCTCCTC-3′ HATF2 probe: 5′-CY5-CCTCTAGATTTATCCCCTCTTGCAACACC-3′ HCREB F: 5′-GCCACAGATTGCCACATTAGC-3′ HCREB R: 5′-TTACGGTGGGAGCAGATGATG-3′ HCREB probe: 5′-CY5-CAGGTATCTATGCCAGCAGCTCATGC-3′ HMEF2 F: 5′-CTCGGACCTCCTCACCTCG-3′ HMEF2 R: 5′-ATGTGCCCGTTGCTGGAC-3′ HMEF2 probe: 5′-CY5-CTGCTCAAGCTGGCGTCGCCC-3′ hACTB F: 5′-GCCCATCTACGAGGGGTATG-3′ hACTB R: 5′-GAGGTAGTCAGTCAGGTCCCG-3′ hACTB probe: 5′-HEX-CCCCCATGCCATCCTGCGTC-3′

### Protein analysis

Total proteins were extracted from cultured cells with cell lysis buffer (60 mMTris-HCl, pH 6.8, 2% SDS, 20% glycerol, 0.25% bromophenol blue, and 1.25% 2-mercaptoethanol) and heated at 95 °C for 10 min. Anti-DLL1 (20230-1-AP) was purchased from San Ying Biotechnology, China (Proteintech). The target proteins were then probed with anti-rabbit IgG peroxidase-conjugated antibody. The target bands were revealed by an enhanced chemiluminescence reaction (Pierce), and the relative density (level) of proteins over the GAPDH band was quantified with the Gel-Pro Analyzer (Media Cybernetics).

### Cell apoptosis analysis

Apoptosis was analyzed using Annexin V/PI double staining. 48 hr after transfection, the cells in the logarithmic growth phase were harvested and rinsed twice with ice-bathed PBS, then 3 μl FITC-labeled enhanced annexin V and 3 μl propidium iodide were added to the 150 μl cell suspension. After incubation for 30 min, flow cytometry was performed on a FACSCalibur instrument. The number of apoptotic and necrotic cells were calculated by flow cytometry (Becton-Dickinson, USA) and analyzed by Flowjo Software. The ratio of early apoptosis was used for the test results. The experiments were performed three times independently, and a representative is shown.

### Luciferase reporter assay

Cells were seeded in 24-well plate at a concentration of 2 × 10^5^ cells/per well and co-transfected 24 hr later with pGL3-luc-Rab27B UTR WT and miR-20a-5p mimic/antagomiR or NC. 48 hr after transfection, cells were collected, and the relative luciferase activity was performed using Dula-Luciferase Reporter Assay Kit (Promega). The relative firefly luciferase activities of the UTR construct was analyzed as previously reported^11^.

### Signaling pathway analysis

The reporter construct encodes the firefly luciferase reporter gene under the control of a basal promoter element (TATA box) joined to tandem repeats of a specific transcriptional response element. The NC construct encodes firefly luciferase under the control of a basal promoter element (TATA box) without any additional transcriptional response elements, is critical for identifying specific effects and determining background reporter activity. The positive control construct is a mix of constructs that constitutively express GFP, firefly luciferase and Renilla luciferase. The cells were transfected in triplicate with each firefly luciferase reporter construct in combination with the Renilla luciferase-based control construct using the riboFECT CP transfection reagent, and both the luciferase activities were measured in the cell extracts 24 hr after transfection. The luciferase activities (luciferase unit) of the pathway reporter relative to those of the negative control in the transfected cells were calculated as a measurement of the pathway activity.

### *In vivo* studies

Four-week BALB/C nude mice were purchased from Silaike Experimental Animal China. All of the animal experiments were carried out in strict adherence with the regulations for the Administration of Affairs Concerning Experimental Animals approved by the State Council of People’s Republic of China. All procedures involving animals and their care in this study were approved and performed by the Institutional Animal Care and Use Committee (IACUC) of the University of Science and Technology of China. The analyzed as previously reported^25^.

### Immunohistochemistry

The expression of DLL1 protein was measured using immunochemical analysis of 5-mm slices of formalin-fixed paraffin-embedded tumor xenografts from the nude mice. The tissue slides from all six groups were placed on a single slide and simultaneously subjected to the same immunestaining. Antigens were retrieved by pretreating dewaxed sections in a microwave oven at 750 watts for 5 min in citrate buffer (pH 6) and processed with the Super Sensitive Link-Labeled Detection System (Biogenex, Menarini, Florence, Italy). The enzymatic activities were determined using 3-amino-9-ethylcarbazole (Dako, Milan, Italy) as a chromogenic substrate. Following counterstaining with Mayer hematoxylin (Invitrogen), the slides were mounted in aqueous mounting medium (glycergel, Dako). Pictures were taken usinga LEICA DM 4000B microscope. Accordingto the rates of positive cell in each field, we marked the chipsas 0 (pigment free), + (light yellow),++(yellow),+++ (51∼75%), and ++++(brownish yellow) by their color intensity^52^.

### Statistical analyses

The data are presented as the mean, and the error bars indicate the S.D. All statistical analyses were performed with Excel (Microsoft, Redmond, WA, USA). Two-tailed Student’s *t*-test, a one-way analysis of variance was used to calculate statistical significance. A *P*-value of <0.05 was considered significant.

### Ethics statement

Animal experiments were undertaken in accordance with the National Institutes of Health Guide for the Care and Use of Laboratory Animals. Animal research was approved by the biomedical ethics committee of Anhui Medical University, when we applying for the National Natural Science Foundation of China (81372868 granted to SBC) in 2013. The animal study proposal was approved by the Institutional Animal Care and Use Committee (IACUC) of the University of Science and Technology of China. All of the mouse experimental procedures were performed in accordance with the Regulations for the Administration of Affairs Concerning Experimental Animals approved by the State Council of People’s Republic of China.

## Additional Information

**How to cite this article:** Pu, Y. *et al*. MiR-34a-5p promotes multi-chemoresistance of osteosarcoma through the down-regulation of the DLL1 gene. *Sci. Rep.* **7**, 44218; doi: 10.1038/srep44218 (2017).

**Publisher's note:** Springer Nature remains neutral with regard to jurisdictional claims in published maps and institutional affiliations.

## Supplementary Material

Supplementary Information

## References

[b1] Pillai R. S., Bhattacharyya S. N. & Filipowicz W. Repression of protein synthesis by miRNAs: how many mechanisms? Trends in cell biology 17, 118–126, doi: 10.1016/j.tcb.2006.12.007 (2007). 17197185

[b2] Lu J. . MicroRNA expression profiles classify human cancers. Nature 435, 834–838, doi: 10.1038/nature03702 (2005). 15944708

[b3] Volinia S. . A microRNA expression signature of human solid tumors defines cancer gene targets. Proceedings of the National Academy of Sciences of the United States of America 103, 2257–2261, doi: 10.1073/pnas.0510565103 (2006). 16461460 PMC1413718

[b4] Allen K. E. & Weiss G. J. Resistance may not be futile: microRNA biomarkers for chemoresistance and potential therapeutics. Molecular cancer therapeutics 9, 3126–3136, doi: 10.1158/1535-7163.MCT-10-0397 (2010). 20940321

[b5] Kalathur R. K. . UniHI 7: an enhanced database for retrieval and interactive analysis of human molecular interaction networks. Nucleic acids research 42, D408–414, doi: 10.1093/nar/gkt1100 (2014). 24214987 PMC3965034

[b6] Gerlinger M. . Intratumor heterogeneity and branched evolution revealed by multiregion sequencing. The New England journal of medicine 366, 883–892, doi: 10.1056/NEJMoa1113205 (2012). 22397650 PMC4878653

[b7] Marin J. J., Briz O., Monte M. J., Blazquez A. G. & Macias R. I. Genetic variants in genes involved in mechanisms of chemoresistance to anticancer drugs. Current cancer drug targets 12, 402–438 (2012). 22229248 10.2174/156800912800190875

[b8] Li F. & Sethi G. Targeting transcription factor NF-kappaB to overcome chemoresistance and radioresistance in cancer therapy. Biochimica et biophysica acta 1805, 167–180, doi: 10.1016/j.bbcan.2010.01.002 (2010). 20079806

[b9] Su S. F. . miR-30d, miR-181a and miR-199a-5p cooperatively suppress the endoplasmic reticulum chaperone and signaling regulator GRP78 in cancer. Oncogene 32, 4694–4701, doi: 10.1038/onc.2012.483 (2013). 23085757 PMC3787795

[b10] Djebali S. . Landscape of transcription in human cells. Nature 489, 101–108, doi: 10.1038/nature11233 (2012). 22955620 PMC3684276

[b11] Lv L. . The DNA methylation-regulated miR-193a-3p dictates the multi-chemoresistance of bladder cancer via repression of SRSF2/PLAU/HIC2 expression. Cell death & disease 5, e1402, doi: 10.1038/cddis.2014.367 (2014). 25188512 PMC4540198

[b12] Schetter A. J. . MicroRNA expression profiles associated with prognosis and therapeutic outcome in colon adenocarcinoma. Jama 299, 425–436, doi: 10.1001/jama.299.4.425 (2008). 18230780 PMC2614237

[b13] Yang L. . Altered microRNA expression in cisplatin-resistant ovarian cancer cells and upregulation of miR-130a associated with MDR1/P-glycoprotein-mediated drug resistance. Oncology reports 28, 592–600, doi: 10.3892/or.2012.1823 (2012). 22614869

[b14] Song B. . Mechanism of chemoresistance mediated by miR-140 in human osteosarcoma and colon cancer cells. Oncogene 28, 4065–4074, doi: 10.1038/onc.2009.274 (2009). 19734943 PMC2783211

[b15] Bommer G. T. . p53-mediated activation of miRNA34 candidate tumor-suppressor genes. Current biology : CB 17, 1298–1307, doi: 10.1016/j.cub.2007.06.068 (2007). 17656095

[b16] Chang T. C. . Transactivation of miR-34a by p53 broadly influences gene expression and promotes apoptosis. Molecular cell 26, 745–752, doi: 10.1016/j.molcel.2007.05.010 (2007). 17540599 PMC1939978

[b17] Corney D. C., Flesken-Nikitin A., Godwin A. K., Wang W. & Nikitin A. Y. MicroRNA-34b and MicroRNA-34c are targets of p53 and cooperate in control of cell proliferation and adhesion-independent growth. Cancer research 67, 8433–8438, doi: 10.1158/0008-5472.CAN-07-1585 (2007). 17823410

[b18] He L. . A microRNA component of the p53 tumour suppressor network. Nature 447, 1130–1134, doi: 10.1038/nature05939 (2007). 17554337 PMC4590999

[b19] Li Y. . MicroRNA-34a inhibits glioblastoma growth by targeting multiple oncogenes. Cancer research 69, 7569–7576, doi: 10.1158/0008-5472.CAN-09-0529 (2009). 19773441 PMC2756313

[b20] Wu J. . MicroRNA-34a inhibits migration and invasion of colon cancer cells via targeting to Fra-1. Carcinogenesis 33, 519–528, doi: 10.1093/carcin/bgr304 (2012). 22198213

[b21] Ji Q. . Restoration of tumor suppressor miR-34 inhibits human p53-mutant gastric cancer tumorspheres. BMC cancer 8, 266, doi: 10.1186/1471-2407-8-266 (2008). 18803879 PMC2564978

[b22] Li N. . miR-34a inhibits migration and invasion by down-regulation of c-Met expression in human hepatocellular carcinoma cells. Cancer letters 275, 44–53, doi: 10.1016/j.canlet.2008.09.035 (2009). 19006648

[b23] Gallardo E. . miR-34a as a prognostic marker of relapse in surgically resected non-small-cell lung cancer. Carcinogenesis 30, 1903–1909, doi: 10.1093/carcin/bgp219 (2009). 19736307

[b24] Botter S. M., Neri D. & Fuchs B. Recent advances in osteosarcoma. Current opinion in pharmacology 16, 15–23, doi: 10.1016/j.coph.2014.02.002 (2014). 24632219

[b25] Pu Y. . MiR-34a-5p promotes the multi-drug resistance of osteosarcoma by targeting the CD117 gene. Oncotarget doi: 10.18632/oncotarget.8546 (2016). PMC5053736 27056900

[b26] Siemens H., Jackstadt R., Kaller M. & Hermeking H. Repression of c-Kit by p53 is mediated by miR-34 and is associated with reduced chemoresistance, migration and stemness. Oncotarget 4, 1399–1415, doi: 10.18632/oncotarget.1202 (2013). 24009080 PMC3824539

[b27] Gao J. . miR-34a-5p suppresses colorectal cancer metastasis and predicts recurrence in patients with stage II/III colorectal cancer. Oncogene 34, 4142–4152, doi: 10.1038/onc.2014.348 (2014). 25362853

[b28] Nakatani F. . miR-34a predicts survival of Ewing’s sarcoma patients and directly influences cell chemo-sensitivity and malignancy. The Journal of pathology 226, 796–805, doi: 10.1002/path.3007 (2012). 21960059

[b29] Misso G. . Mir-34: a new weapon against cancer? Molecular therapy. Nucleic acids 3, e194, doi: 10.1038/mtna.2014.47 (2014). PMC4222652 25247240

[b30] Pang R. T. . MicroRNA-34a suppresses invasion through downregulation of Notch1 and Jagged1 in cervical carcinoma and choriocarcinoma cells. Carcinogenesis 31, 1037–1044, doi: 10.1093/carcin/bgq066 (2010). 20351093

[b31] Kastl L., Brown I. & Schofield A. C. miRNA-34a is associated with docetaxel resistance in human breast cancer cells. Breast cancer research and treatment 131, 445–454, doi: 10.1007/s10549-011-1424-3 (2012). 21399894

[b32] Fujita Y. . Effects of miR-34a on cell growth and chemoresistance in prostate cancer PC3 cells. Biochemical and biophysical research communications 377, 114–119, doi: 10.1016/j.bbrc.2008.09.086 (2008). 18834855

[b33] Kojima K., Fujita Y., Nozawa Y., Deguchi T. & Ito M. MiR-34a attenuates paclitaxel-resistance of hormone-refractory prostate cancer PC3 cells through direct and indirect mechanisms. The Prostate 70, 1501–1512, doi: 10.1002/pros.21185 (2010). 20687223

[b34] Koch U. & Radtke F. Notch and cancer: a double-edged sword. Cellular and molecular life sciences : CMLS 64, 2746–2762, doi: 10.1007/s00018-007-7164-1 (2007). 17687513 PMC11136344

[b35] Iso T., Kedes L. & Hamamori Y. HES and HERP families: multiple effectors of the Notch signaling pathway. Journal of cellular physiology 194, 237–255, doi: 10.1002/jcp.10208 (2003). 12548545

[b36] Grabher C., von Boehmer H. & Look A. T. Notch 1 activation in the molecular pathogenesis of T-cell acute lymphoblastic leukaemia. Nature reviews. Cancer 6, 347–359, doi: 10.1038/nrc1880 (2006). 16612405

[b37] Dang T. P. . Chromosome 19 translocation, overexpression of Notch3, and human lung cancer. Journal of the National Cancer Institute 92, 1355–1357 (2000). 10944559 10.1093/jnci/92.16.1355

[b38] Park J. T. . Notch3 gene amplification in ovarian cancer. Cancer research 66, 6312–6318, doi: 10.1158/0008-5472.CAN-05-3610 (2006). 16778208

[b39] Miyamoto Y. . Notch mediates TGF alpha-induced changes in epithelial differentiation during pancreatic tumorigenesis. Cancer cell 3, 565–576 (2003). 12842085 10.1016/s1535-6108(03)00140-5

[b40] Engin F. . Notch signaling contributes to the pathogenesis of human osteosarcomas. Human molecular genetics 18, 1464–1470, doi: 10.1093/hmg/ddp057 (2009). 19228774 PMC2733809

[b41] Zhang P., Yang Y., Nolo R., Zweidler-McKay P. A. & Hughes D. P. Regulation of NOTCH signaling by reciprocal inhibition of HES1 and Deltex 1 and its role in osteosarcoma invasiveness. Oncogene 29, 2916–2926, doi: 10.1038/onc.2010.62 (2010). 20208568 PMC2874642

[b42] Hughes D. P. How the NOTCH pathway contributes to the ability of osteosarcoma cells to metastasize. Cancer treatment and research 152, 479–496, doi: 10.1007/978-1-4419-0284-9_28 (2009). 20213410

[b43] Dailey D. D. . HES1, a target of Notch signaling, is elevated in canine osteosarcoma, but reduced in the most aggressive tumors. BMC veterinary research 9, 130, doi: 10.1186/1746-6148-9-130 (2013). 23816051 PMC3701487

[b44] Tanaka M. . Inhibition of Notch pathway prevents osteosarcoma growth by cell cycle regulation. British journal of cancer 100, 1957–1965, doi: 10.1038/sj.bjc.6605060 (2009). 19455146 PMC2714252

[b45] Montojo J., Zuberi K., Rodriguez H., Bader G. D. & Morris Q. GeneMANIA: Fast gene network construction and function prediction for Cytoscape. F1000Research 3, 153, doi: 10.12688/f1000research.4572.1 (2014). 25254104 PMC4168749

[b46] Deng H. . The miR-193a-3p regulated PSEN1 gene suppresses the multi-chemoresistance of bladder cancer. Biochimica et biophysica acta 1852, 520–528, doi: 10.1016/j.bbadis.2014.12.014 (2015). 25542424

[b47] Su Y. . Establishment and characterization of a new highly metastatic human osteosarcoma cell line. Clinical & experimental metastasis 26, 599–610, doi: 10.1007/s10585-009-9259-6 (2009). 19363654

[b48] Tarazona S., Garcia-Alcalde F., Dopazo J., Ferrer A. & Conesa A. Differential expression in RNA-seq: a matter of depth. Genome research 21, 2213–2223, doi: 10.1101/gr.124321.111 (2011). 21903743 PMC3227109

[b49] Heiser L. M. . Subtype and pathway specific responses to anticancer compounds in breast cancer. Proceedings of the National Academy of Sciences of the United States of America 109, 2724–2729, doi: 10.1073/pnas.1018854108 (2012). 22003129 PMC3286973

[b50] Andrisano V., Bartolini M., Gotti R., Cavrini V. & Felix G. Determination of inhibitors’ potency (IC50) by a direct high-performance liquid chromatographic method on an immobilised acetylcholinesterase column. Journal of chromatography. B, Biomedical sciences and applications 753, 375–383 (2001). 11334353 10.1016/s0378-4347(00)00571-5

[b51] Liu K. . MIR34A regulates autophagy and apoptosis by targeting HMGB1 in the retinoblastoma cell. Autophagy 10, 442–452, doi: 10.4161/auto.27418 (2014). 24418846 PMC4077883

[b52] Tu L. . Correlations of fascin-1 and cadherin-17 protein expression with clinicopathologic features and prognosis of patients with gastric cancer. Tumour biology : the journal of the International Society for Oncodevelopmental Biology and Medicine 37, 8775–8782, doi: 10.1007/s13277-015-4368-0 (2016). 26743780

