# Supplementary material for: MiR-34a-5p promotes multi-chemoresistance of osteosarcoma through down-regulation of the DLL1 gene
Source: Sci Rep. 2017 Mar 10;7:44218. doi: 10.1038/srep44218 (PMC5345075; doi:10.1038/srep44218)
Supplement: Supplementary Information [file srep44218-s1.pdf]

MiR-34a-5p promotes multi-chemoresistance of osteosarcoma through the down-regulation of the DLL1 gene

Youguang Pu, Fangfang Zhao, Haiyan Wang & Shanbao Cai

Additional File 1

A

N0.	miR_name	SJSA-1	G-292	MG63.2	SJSA-1/G-292	SJSA-1/MG63.2
1	hsa-miR-432-5p	2036	6	9	339.33	226.22
2	hsa-miR-143-3p	1543	37	137	41.70	11.26
3	hsa-miR-26a-5p	23063	1677	1459	13.75	15.81
4	hsa-miR-199a-3p	30480	3641	46351	8.37	0.66
5	hsa-miR-199b-3p	30478	3641	46349	8.37	0.66
6	hsa-miR-199a-5p	58	8	119	7.25	0.49
7	hsa-miR-193b-5p	1976	275	1069	7.19	1.85
8	hsa-miR-193b-3p	314	54	607	5.81	0.52
9	hsa-miR-214-3p	297	56	108	5.30	2.75
10	hsa-miR-98-5p	827	211	734	3.92	1.13
11	hsa-let-7f-5p	803209	207849	732306	3.86	1.10
12	hsa-miR-4421	27	7	20	3.86	1.35
13	hsa-miR-532-5p	100	26	183	3.85	0.55
14	hsa-miR-450a-2-3p	26	7	7	3.71	3.71
15	hsa-let-7b-5p	823266	222152	1055658	3.71	0.78
16	hsa-miR-365a-5p	492	133	567	3.70	0.87
17	hsa-miR-1287-5p	28	8	41	3.50	0.68
18	hsa-miR-34a-5p	157	46	2	3.41	78.50
19	hsa-miR-6758-5p	45	14	6	3.21	7.50
20	hsa-miR-152-3p	1093	404	5526	2.71	0.20
21	hsa-miR-1255b-5p	15	6	10	2.50	1.50
22	hsa-miR-335-5p	40	17	0	2.35	0
23	hsa-miR-452-5p	985	422	312	2.33	3.16
24	hsa-let-7d-3p	162	71	207	2.28	0.78
25	hsa-miR-3179	13	6	16	2.17	0.81
26	hsa-miR-642a-3p	30	14	4	2.14	7.50
27	hsa-let-7i-5p	15762	7838	26939	2.01	0.59
28	hsa-miR-200c-3p	26	13	12	2.00	2.17

B

No.	Symbol	SJSA-1	G-292	MG63.2	G-292/SJSA-1	MG63.2/SJSA-1
1	PRKCQ	0.01	5.46	0.19	545.88	19.32
2	AGTR1	0.01	4.90	0.54	490.16	53.84
3	DLL1	0.01	1.98	3.62	197.60	362.16
4	NUP210	0.14	22.59	6.66	155.90	45.96
5	RPS6KL1	0.01	1.51	0.50	151.31	50.28
6	PPP1R16B	0.01	1.26	0.00	125.75	0.00
7	KIT	0.01	1.09	0.84	108.73	83.89
8	UBE2QL1	0.01	0.92	0.00	92.08	0.00
9	ADRA1D	0.01	0.38	1.22	37.80	122.48
10	BTBD11	0.36	10.88	1.89	30.08	5.22
11	TPPP	0.03	0.97	0.50	28.05	14.62
12	CBFA2T3	0.01	0.28	1.29	27.66	129.45
13	E2F5	0.48	12.14	2.12	25.39	4.44
14	CTNND2	0.01	0.22	0.32	22.23	32.01
15	MGAT4A	0.02	0.38	0.10	15.47	4.18
16	CSRNP3	0.01	0.15	0.09	15.39	9.23
17	ACVR2B	0.33	4.34	0.48	13.16	1.45
18	SATB1	0.44	5.35	0.08	12.09	0.17
19	ANK3	0.16	1.67	6.14	10.49	38.60
20	FUT1	0.10	0.85	0.21	8.70	2.09
21	FAM83A	0.07	0.62	0.00	8.70	0.00

The interested miRNA and mRNA genes based on the websites and RNA-seq analysis. A dozen of miRNAs were differentially expressed in the multi-chemoresistant OS cells SJSA-1 and the multi-chemosensitive OS cells G-292 and MG63.2 based on the websites, and the ratio over 2 of SJSA-1/G-292 based on RNA-seq-based miR-omic analysis were showed in descending order, has-miR-34a-5p was one of them (**A**). Reference to similar methods, the downstream genes of miR-34a-5p were also showed, the ratio of G-292/SJSA-1 based on RNA-seq analysis were showed in descending order, DLL1 is located (**B**).

Additional File 2

A

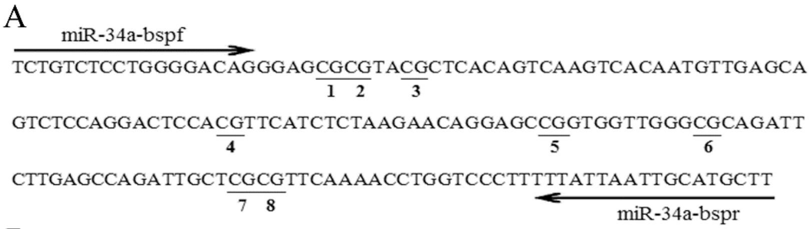

B

	No.	CpGSite	Base_calls	Base	C/(C+T)	CpGSite	Base_calls	Base	C/(C+T)	Average
		Forward				Reverse				
G-292	1					23	329	C	86.90	86.90
	2					25	353	C	84.48	84.48
	3	29	410	C	86.65	29	400	T	72.23	79.44
	4	73	674	C	72.27	73	907	T	43.66	57.97
	5	96	806	C	82.85	96	1161	A	5.24	44.05
	6	107	1077	C	84.29					84.29
	7	131	1099	C	99.29					99.29
	8									
	Average				85.07				58.50	76.63

C

	No.	CpGSite	Base_calls	Base	C/(C+T)	CpGSite	Base_calls	Base	C/(C+T)	Average
		Forward				Reverse				
SJSA−1	1					23	331	C	87.49	87.49
	2					25	356	C	69.56	69.56
	3	29	413	T	61.76	29	402	T	67.15	64.46
	4	73	675	C	74.72	73	912	T	62.04	68.38
	5	96	807	C	60.17	96	1173	A	8.68	34.43
	6	107	1076	C	75.92					75.92
	7	131	1099	C	88.05					88.05
	8									
	Average				72.124				58.98	69.75

Differential methylation of the miR-34a gene in SJSA-1 cells compared with G-292 cells. BSP primers and CpGdinucleotides of miR-34a were shown (A). Methylation percentage at each CpG site in the SJSA-1 and G-292 cells (B).
